# Implant Surface Topography Following Different Laser Treatments: An In Vitro Study

**DOI:** 10.7759/cureus.38731

**Published:** 2023-05-08

**Authors:** Marwa I Khalil, Haitham Sakr

**Affiliations:** 1 Department of Oral Medicine, Periodontology, Oral Diagnosis, and Oral Radiology, Faculty of Dentistry, Alexandria University, Alexandria, EGY; 2 Department of Oral and Maxillofacial Surgery, Faculty of Dentistry, Pharos University in Alexandria, Alexandria, EGY

**Keywords:** er:cr:ysgg laser, diode laser, topography, surface roughness, laser treatment

## Abstract

Background

Although dental implants have demonstrated very high success rates, they are susceptible to complications such as peri-implantitis that can lead to failure.

Methods

Twenty implants with surfaces grit-blasted using hydroxyapatite and acid-etched were randomly divided into four groups (five in each group). Three groups were assigned to laser treatments: Group I (erbium, chromium-doped: yttrium, scandium, gallium, and garnet (Er,Cr:YSGG) laser), Group II (650-nm diode laser), and Group III (808-nm diode), and one control group, Group IV. The surface roughness parameters (roughness average(Ra) and root mean square roughness(Rq)) were measured using a non-contact optical profilometer and scanning electron microscope to evaluate the surface topography after the laser treatments.

Results

Significant differences were observed between the laser groups regarding the surface roughness Ra (3.56±0.26, 3.45±0.19, 3.77±0.42, p_c_=0.0004, p_e_=0.0002, p_f_=0.001) and Rq values (4.49±0.34, 4.35±0.26, 4.72±0.56, p_c_=0.0007, p_e_=0.0006, p_f_=0.002) and the control group (2.81±0.10; 3.57±0.19). However, no significant difference was observed between the different laser treatment modalities. The scanning electron microscope images revealed some morphological changes on the implant surfaces following laser treatment, but no melted morphology was observed.

Conclusions

The application of Er,Cr:YSGG, 650-nm diode laser, and 808-nm diode did not show melting changes on implant topography. However, some increase in surface roughness was detected. Further studies are recommended to assess the effectiveness of these laser settings on bacterial reduction and osseointegration.

## Introduction

Although dental implants have demonstrated very high success rates, they are susceptible to complications that can lead to failure [1]. Among these complications is peri-implantitis, an inflammatory condition of the tissue surrounding the implant that causes bone loss and ultimately results in the loss of the implant [2].

Decontaminating dental implants effectively is challenging, and numerous methods have been reported for their management [3]. Various methods have been used to treat peri-implantitis, including mechanical debridement and chemical surface treatments, but their effectiveness has been limited and may have caused further damage to the implant [4,5]. Additionally, laser therapy has been investigated as a potential treatment for peri-implant disease [6]. Laser irradiation can be effective in reducing the accumulation of bacteria on the implant surface and decontaminates it to prevent peri-implantitis [7].

The surface topography of titanium implants includes features ranging from macro to nanoscale, designed to promote osseointegration, thereby enhancing the long-term stability of the interface between the implant surface and the surrounding bone. Despite the efficacy of lasers in treating peri-implantitis, the question remains of whether direct laser application changes the surface of titanium, and if so, its impact on the surface properties [8]. Different laser parameters and wavelengths have been found in several studies to have different effects on implant surfaces [8,9].

Since the parameters of lasers and implant systems can be combined in different ways, the literature in this regard shows conflicting findings. The direct absorption of laser energy in the implant surface as well as the bacterial biofilm [10] can lead to the degradation and alteration of the implant surface topography [11].

The member of the erbium laser family, erbium chromium-doped: and yttrium, scandium, gallium, and garnet (Er,Cr:YSGG) has a wavelength of 2,780 nm. In addition to treating peri-implantitis, this laser is effective in eliminating biofilms from the surface of implants [12,13]. However, applying high-intensity pulses for a short duration may cause changes to titanium surfaces [14].

Diode lasers have been successfully used for the treatment of peri-implantitis due to their photothermal decontaminating properties. Several wavelengths of these lasers exist, including gallium aluminum arsenide lasers (650 to 830 nm), gallium arsenide lasers (810 to 840 nm), and indium aluminum arsenide lasers (904 to 980 nm) [15].

An important factor affecting osseointegration is the roughness of the dental implant surface or micro-topography. The ideal surface roughness of dental implants is defined by a microtopographic profile of 1-10 micrometers. Within this range of roughness, the surface of the implant is most likely to interlock with mineralized bone [16,17]. Several studies have shown that a moderately rough implant surface, achieved by sandblasting with acid-etching, promotes osteoblastic cell differentiation and promotes faster osseointegration [18,19].

A high success rate has been reported for moderately rough implants compared to machined or smoother ones, especially in patients with reduced bone quality [18]. According to Sammons et al., the microstructured titanium surface treatment produced by acid-etching and sandblasting could enhance the spread of rat osteoblasts [20].

Thus, the purpose of this study was to investigate and compare the effects of different dental lasers on implant surface topography.

## Materials and methods

On the basis of previous studies [21,22], sample sizes were estimated for each group based on five samples. The study approval was obtained from the Unit of Research Ethics Approval Committee of Pharos University, Alexandria, Egypt (approval number: 26-2-2023/3-059). Twenty commercially available micro-textured treatment implants (Xive-implants, 3.8 x 11 mm; Dentsply Sirona, Charlotte, North Carolina, United States) with surfaces grit-blasted using hydroxyapatite and acid-etched were randomly divided into four groups (five in each group) and assigned to different treatments (Table 1).

**Table 1:** Different tested groups with laser settings used in the study Er,Cr:YSGG: Erbium, Chromium-doped: Yttrium-Scandium-Gallium-Garnet

Groups	Laser treatment	Laser parameters	Duration
Group I	Er,Cr:YSGG (2780 nm H mode)	1 W, 30 Hz, 120 mJ air and water 50% contact mode	60 s/ surface
Group II	650 diode	50 mW, 60 mJ non-contact mode	60 s/ surface
Group III	808 diode	1W, 120 mJ non-contact mode	60 s/ surface
Group IV	No treatment	-	-

The four groups with five implants in each were divided as follows: (i) Group I: Laser irradiation was performed using an Er:Cr:YSGG 2780 nm laser (Waterlase iPlus; BIOLASE, Inc., California, United States) emitting an axial and radial laser beam at a wavelength of 2,780 mm. A short-pulse H-mode irradiation was carried out with a setting of 50% water and 50% air, a frequency of 30 Hz, and an intensity of 1 W and 120 mJ. A custom-made mount held the MZ8-6mm (ZipTip) fiber tip perpendicular to the implant surface at a distance of 0.5 mm; (ii) Group II: Using a gallium aluminum arsenide (GaAlAs) diode laser operating at wavelength of 650 nm (Lasercat 500; MedSolution, Radolfzell, Germany), the implant surfaces were irradiated at a distance of 0.5 mm with an output power of 50 mW; (iii) Group III: The treatment was conducted using a diode laser (Lasercat 500; MedSolution) operating at a wavelength of 808 nm at a distance of 0.5mm (1 W), (iv) Group IV: No surface treatment was performed.

For standardization, only one investigator performed the laser application to the implant surface. A blinded statistician provided coding labels to the containers with implants in each group. Surface roughness measurements for the coded implant groups were performed by two investigators blinded to the codes provided. Measurements were performed three times by each investigator and repeated after two weeks to assess intra-examiner reliability.

Surface roughness

The roughness average (Ra) was the arithmetic average of the absolute values of the profile heights over the evaluation length. The root mean square roughness (Rq) was the root mean square of the values of all points of the profile. Both were measured using a non-contact optical profilometer (ContourGT-I 3D Optical Microscope; Bruker Corporation, Billerica, Massachusetts, United States) before the decontamination procedures. Readings were detected for each specimen three times on the lateral flat surface of the apical third of each implant at a speed of 0.5 mm per second, and the cutoff was set as 0.8 mm. The mean of these readings was then calculated.

Scanning electron microscopy (SEM) evaluation

Two samples from each group were subjected to SEM evaluation to study the topographical changes in the implant surfaces following the laser treatment. The implants were mounted on aluminum stubs with carbon adhesive tabs, electrically grounded with colloidal graphite, and sputter-coated for six minutes with gold-palladium (Hummer™6.2; Anatech USA, Sparks, New Virginia, United States). The implants were imaged in an SEM (Inspect S50; FEI Company, Hillsboro, Oregon, United States), which was operated at 20 kV, and electronic images were captured. Four images were then recorded from each implant at the apical threads using 6,000x and 12,000x magnification.

Statistical analysis

The intra- and inter-examiner agreements were assessed using Cohen's kappa coefficient. The means and standard deviations of the surface roughness values and surface parameters obtained by both examiners were calculated and analyzed using SPSS for Windows, Version 14.0 (SPSS Inc., Chicago, Illinois, United States). Non-parametric Kruskal-Wallis, Mann-Whitney, and Friedman tests were used to compare the differences between the groups. P-values of <0.05 were considered statistically significant.

## Results

The intra- and inter-examiner agreement showed a κ-coefficient of 0.98 and 0.97, respectively. Statistical analysis was conducted using the average of both examiners' scores.

Surface parameters

**Figure 1:**
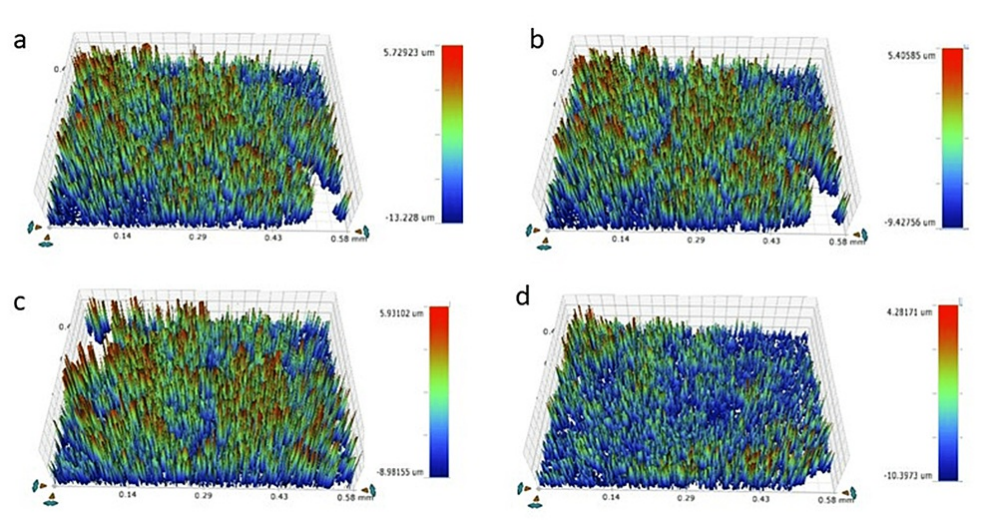
Surface roughness (Ra) of (a) Group I (Er,Cr: YSGG), (b) Group II (650nm diode laser), (c) Group III (808nm diode laser), (d) Group IV (no laser treatment) Er,Cr:YSGG: Erbium, Chromium-doped: Yttrium-Scandium-Gallium-Garnet

The Ra and Rq results are presented in Table 2 and Figure 1. Significant differences were observed in the Ra and Rq values between groups I, II, III, and IV (no treatment). However, no significant difference was observed between the different laser treatment groups.

**Table 2:** Quantitative values obtained for different surface parameters for the studied groups. *Significant difference* P* <0.05 *P_a_*: between groups I and II,* P_b_:* between groups I and III, *P_c_* : between groups I and IV, *P_d_*: between groups II and III, *P_e_*: between groups II and IV, and *P_f _*: between groups III and IV Er,Cr:YSGG: Erbium, Chromium-doped: Yttrium-Scandium-Gallium-Garnet

Parameters	Group I (Er,Cr: YSGG), mean± SD	Group II (650 Diode), mean± SD	Group III (808 Diode), mean± SD	Group IV (No treatment), mean± SD	p-value
Ra	3.56±0.26	3.45±0.19	3.77±0.42	2.81±0.10	P_a _=0.4595, P_b _=0.3771, P_c _=0.0004,* P_d _=0.1589, P_e _=0.0002*, P_f _=0.0011*
Rq	4.49±0.34	4.35±0.26	4.72±0.56	3.57±0.19	P_a_ =0.4854, P_b_ = 0.4551, P_c_ =0.0007*, P_d_ = 0.2171, P_e_ =0.0006*, P_f_ =0.0025*

SEM analysis

**Figure 2:**
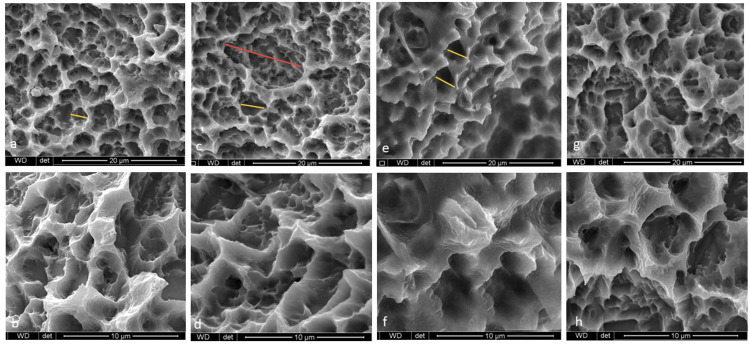
SEM micrographs of implant surfaces showing the porous structures on the micron scale that are caused by acid etching, overlaid by macroporous structures caused by gritblasting: (a, b) Group I: Er,Cr: YSGG, titanium surface, (c, d) Group II: 650nm diode laser, (e, f) Group III: 808nm diode laser, (g, h) Group IV: no laser treatment group (Magnification 6000X and 12000X, respectively) The laser groups (Groups I-III) had larger macropores (red arrows) and more pitted micropores (yellow arrows) SEM: Scanning Electron Microscopy; Er,Cr:YSGG: Erbium, Chromium-doped: Yttrium-Scandium-Gallium-Garnet

The SEM images showed a rough and irregular honeycomb surface, which is characteristic of grit-blasted and acid-etched surface implants [23]. The images showed several micron-sized porous structures formed by acid etching, overlayed by macropores formed by grit-blasting. Some morphological changes were observed on the implant surfaces following laser treatment presented as an increase in the macropores diameter (red arrow) and more pitted micro-pores (yellow arrows) than the control group. However, no melted morphology was observed (Figure 2).

## Discussion

An increase in laser duration or power setting can destroy residual bacterial biofilms; however, this would change the surface topography of the implants. In this study, Er,Cr:YSGG and 808 nm diode lasers (1 W and 120 J ) and 650 nm diode laser (50 mW and 60 mJ) were used for 60 seconds per implant surface. These selected settings were within the range of those commonly used in clinical practice (7-18 J/cm^2^ and 0.5-2 W) [15]. 

The use of mechanical and chemical methods of cleaning and decontaminating implant surfaces has been recommended [24,25]. The wound healing process may be compromised if implant decontamination is inadequate following treatment for peri-implantitis [26]. A laser has been recommended to sterilize and clean implant surfaces [27-28]. Understanding the effects of lasers on titanium implant surfaces is essential given their widespread use for detoxification. Er,Cr:YSGG laser is efficient in removing contamination from implant bodies. In contrast, other laser systems work in vaporization mode, and the resultant elevated temperatures may result in implant surfaces that are damaged and tissue that is charred or coagulated [13].

Laser irradiation of titanium implants has been associated with limited data. In addition, no clear guidelines have been developed for the irradiation of dental implants, including the different settings available.

In a previous study [6], Er,Cr:YSGG laser was used to irradiate titanium discs at 125 or 190 J/cm^2^ and in an air and water mixture of 45% and 20%, respectively [6]. The irradiation distance was approximately 2 mm. Following laser treatment, the specimen surfaces displayed a locally melted morphology. Increasing energy resulted in a slight increase in the melting area and surface roughness.

A commercially available implant was used in this study in order to replicate the clinical setting where the laser beam is directed toward the threads of the implant rather than a flat disc [14]. The effect of implant macrostructure was taken into account since it may have an impact on the outcomes. 

A study by Park et al. used power of 1-5 W and a 20-Hz frequency to irradiate titanium discs for 30 seconds [14]. No change occurred in the surfaces that were irradiated with 1 W and 2 W. At 3 W, 4 W, and 5 W, the surface was altered by melting, coagulation, and microfractures. The authors concluded that the surface damage levels were correlated with elevating the power.

Similarly, in the current study, Er,Cr:YSGG laser irradiation from a distance of 0.5 mm at 1 W altered the roughness of the implant surface. Compared to other laser groups, no significant changes occurred. However, the changes were significant compared with non-treated implant surfaces. Energy dose and duration are important parameters that should be considered during the irradiation process because they greatly influence surface topography [14].

Azzeh et al. decontaminated implant surfaces using a laser in non-contact mode at 1.75 W at 20 Hz with 15% water and 30% air at a distance of 5 mm [29]. The handpiece was oriented perpendicular to the implant surface to ensure that the surface was not altered. The present study demonstrated that parameters of 50% water and 50% air, 30 HZ, 1 W, and 120 J at a 0.5-mm distance did not adversely affect the implant surface topography. The surface roughness values were increased, but the SEM images showed the preservation of the acid-etched and grit-blasted appearance of micron-sized pores overlayed by macropores.

The short time interval (60 seconds) of irradiation used in this study may have contributed to the minimal increase (1 µm) in surface roughness. Similarly, Park et al. found that Er,Cr:YSGG laser at 1 W or 2 W of power did not alter either machined or anodized titanium surfaces [14]. However, a power of 3 W showed severe surface alterations.

Research has demonstrated that roughening the implant surface enhances hydrophilicity and stimulates bone growth, resulting in direct apposition of bone to the titanium implant [20]. Following laser treatments, the mean roughness values ranged from 3.45 µm to 3.77 µm, which still represents a rough implant surface [30].

The limitation of the current study is that no bacterial contamination was performed to test the decontamination effect of these lasers with the selected settings. Moreover, other implants with different surface treatments should be used to assess the effects of these parameters on different surface treatments.

## Conclusions

In this in vitro study, the application of Er,Cr:YSGG, 650-nm, and 808-nm diode lasers for 60 seconds on the grit-blasted and acid-etched implant resulted in an increase in surface roughness up to 1 µm. There was an increase in micro-pores as observed under a scanning electron microscope, however, there was no evidence of surface melting.

A laser application of 1 W for a period of 60 seconds can be used for implant surface treatment without compromising the surface topography. The effectiveness of these settings for implant decontamination should be examined in future studies.
